# Lysosomes’ fallback strategies: more than just survival or death

**DOI:** 10.3389/fcell.2025.1559504

**Published:** 2025-03-11

**Authors:** Quan Wang, Ruolin Wang, Haihui Hu, Xiaoqing Huo, Fulong Wang

**Affiliations:** ^1^ Key Laboratory of Developmental Genes and Human Disease, School of Life Science and Technology, Southeast University, Nanjing, China; ^2^ Huaian Maternity and Child Healthcare Hospital of JiangSu Province, Huaian, China

**Keywords:** lysosomal damage, secretion, exosome, autophagy, golgi apparatus, endoplasmic reticulum

## Abstract

Lysosomes are heterogeneous, acidic organelles whose proper functionality is critically dependent on maintaining the integrity of their membranes and the acidity within their lumen. When subjected to stress, the lysosomal membrane can become permeabilized, posing a significant risk to the organelle’s survival and necessitating prompt repair. Although numerous mechanisms for lysosomal repair have been identified in recent years, the progression of lysosome-related diseases is more closely linked to the organelle’s alternative strategies when repair mechanisms fail, particularly in the contexts of aging and pathogen infection. This review explores lysosomal responses to damage, including the secretion of lysosomal contents and the interactions with lysosome-associated organelles in the endolysosomal system. Furthermore, it examines the role of organelles outside this system, such as the endoplasmic reticulum (ER) and Golgi apparatus, as auxiliary organelles of the endolysosomal system. These alternative strategies are crucial to understanding disease progression. For instance, the secretion and spread of misfolded proteins play key roles in neurodegenerative disease advancement, while pathogen escape via lysosomal secretion and lysosomotropic drug expulsion underlie cancer treatment resistance. Reexamining these lysosomal fallback strategies could provide new perspectives on lysosomal biology and their contribution to disease progression.

## Introduction

**FIGURE 1:**
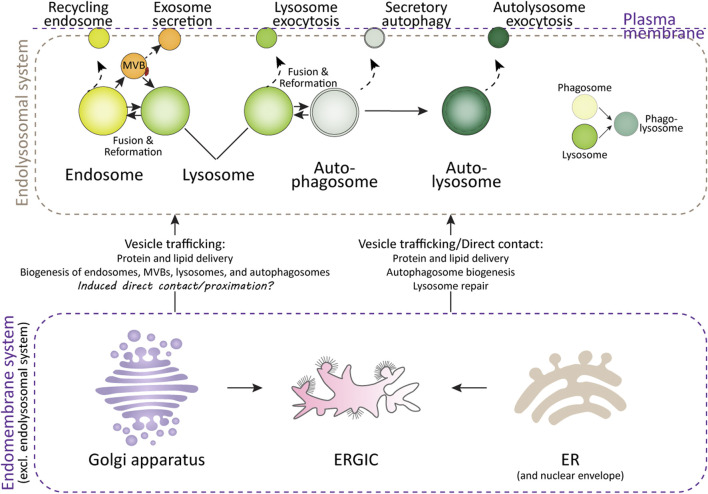
Lysosomes and their interacting organelles. Top panel: Overview of lysosomes and their interacting organelles within the endolysosomal system. Lysosomal fusion and fission with endosomes, autophagosomes, and phagosomes are critical for their maturation, functional maintenance, reformation, and repair after damage. However, when lysosomal damage exceeds the capacity for repair, both lysosomes and the organelles within the endolysosomal system may undergo secretion in various forms. Bottom panel: Interaction of lysosomes with organelles in the endomembrane system. The delivery of proteins and lipids from the ER and Golgi apparatus is essential for lysosomal function and repair. Additionally, the ER, ER-Golgi intermediate compartment (ERGIC), and Golgi apparatus contribute to autophagosome formation, a process critical for the clearance of damaged lysosomes.

Lysosomes are the primary degradation organelles of the cell and serve as the terminal compartments within the endolysosomal system (Ballabio and Bonifacino, 2020). This system comprises a network of dynamic and interconnected organelles, including endosomes, multivesicular bodies (MVBs), lysosomes, lysosome-related organelles (e.g., granules in immune cells), phagosomes, and autophagosomes (Figure 1). From a spatiotemporal perspective, rather than functioning as isolated compartments, the endolysosomal system operates as a highly coordinated and adaptive “digestive tract,” facilitating the sequestration, sorting, and degradation of cargo derived from both intracellular and extracellular sources (Hu et al., 2015; Neel et al., 2024). Endosomes, phagosomes, and autophagosomes act as intermediary hubs within this network, directing cargo toward lysosomes, which serve as the terminal degradative compartments where cellular digestion and recycling occur. Beyond their degradative role, components of this system are involved in specialized secretion pathways (Figure 1): autophagosomes facilitate secretory autophagy, MVBs mediate the release of exosomes, and lysosomes participate in lysosome-associated exocytosis, thereby expelling undigested material, especially when the degradation function of the lysosomes is compromised (Buratta et al., 2020; Han et al., 2022; Ponpuak et al., 2015).

From an endolysosome-centric perspective, other organelles within the broader endomembrane system—such as the ER, Golgi apparatus, and plasma membrane—serve as auxiliary organelles or components of the endolysosomal system (Figure 1). These organelles support lysosomal function by synthesizing and delivering lipids, proteins, and membranes via vesicular trafficking, membrane budding, and direct membrane contact sites. These interactions ensure the proper functioning of the endolysosomal system and maintain cellular homeostasis (De Tito et al., 2020; Nascimbeni et al., 2017; Sun et al., 2024; Tan and Finkel, 2023).

Therefore, when lysosomes are exposed to various stresses and their membrane integrity is compromised, organelles within the endolysosomal and endomembrane systems may play critical roles in facilitating lysosomal repair. These stresses can arise from physiological, pathological, and external factors that impair membrane integrity and enzymatic function. Physiologically, aging contributes to lysosomal damage by promoting the accumulation of indigestible materials, such as lipofuscin, which reduces degradation efficiency, while oxidative stress induces lipid peroxidation within lysosomal membranes, weakening their structure and leading to enzyme leakage (Gahlot et al., 2024; Pan et al., 2021; Zhang et al., 2023). Pathological conditions—including neurodegenerative diseases (Root et al., 2021), lysosomal storage disorders (Parenti et al., 2021), metabolic disturbances (Almeida et al., 2020), and infections (Richards et al., 2022)—exacerbate these effects by causing substrate overload, protein aggregation, and inflammation, further destabilizing lysosomes. Additionally, external agents such as lysosomotropic drugs, ionophores, toxins, and environmental pollutants can directly damage lysosomal membranes, triggering the excessive release of lysosomal enzymes. In research settings, compounds like L-leucyl-L-leucine methyl ester (LLOMe), Glycyl-L-phenylalanine 2-naphthylamide (GPN), methyl-serine dodecylqamide hydrochloride (MSDH), chloroquine, Bafilomycin A1, ammonium chloride, silica crystals, and overexpression of mutant proteins associated with neurodegenerative diseases and lysosomal storage disorders—such as alpha-synuclein, amyloid-beta, Tau, Huntingtin, TDP43, SOD1, PANK2, NPC1, NPC2, CLN3, GBA1, HEXA, GAA, MPS, ASAH1, CTNS, and GALC—are commonly used to mimic these conditions (Parenti et al., 2021; Root et al., 2021; Udayar et al., 2022). Pathogen-derived factors, such as SapM, PtpA, and ESAT-6 from *Mycobacterium tuberculosis* (Ramon-Luing et al., 2023); Listeriolysin O (LLO) from *Listeria monocytogenes* (Shaughnessy et al., 2006); SopB from *Salmonella enterica* (Bakowski et al., 2010); Nef from HIV (Sanfridson et al., 1997); and ORF3a from SARS-CoV-2 (Walia et al., 2024), are also employed to study lysosomal dysfunction.

To date, a wide array of key proteins and complexes have been identified as being recruited to the damaged lysosomal sites to mediate repair processes. These include the Endosomal Sorting Complexes Required for Transport (ESCRT) machinery (Jia et al., 2020b; Radulovic et al., 2018; Skowyra et al., 2018), Annexins (Ebstrup et al., 2023; Yim et al., 2022), mTOR (Jia et al., 2018; Jia et al., 2019), AMPK (Jia et al., 2020a), PI4K2A (Tan and Finkel, 2022), and stress granules (Bussi et al., 2023; Duran et al., 2024; Jia et al., 2022). However, the successful execution of lysosomal repair or resolution of lysosomal stress is heavily dependent on the collaboration and interactions with other organelles. This includes fusion and fission events involving lysosomes, endosomes, and autophagosomes (Bhattacharya et al., 2023; Maejima et al., 2013; Rodgers et al., 2022; Saffi and Botelho, 2019), as well as both direct and indirect interactions with the ER (Radulovic et al., 2022; Tan and Finkel, 2022; Wang et al., 2024), Golgi apparatus (Lie et al., 2021; Vitry et al., 2010), and plasma membrane (Domingues et al., 2024; Sho et al., 2024; Wang et al., 2023; Zhong et al., 2023) (Figure 1).

**FIGURE 2:**
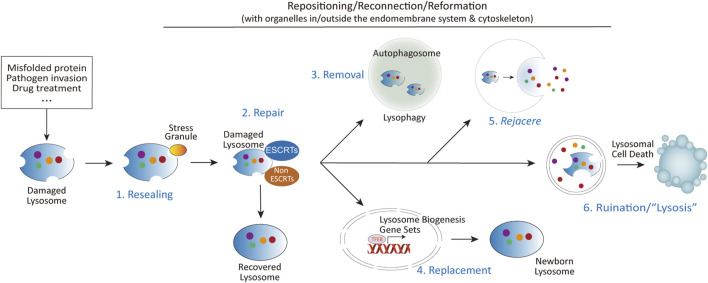
The diverse fates of damaged lysosomes. Upon lysosomal damage caused by aggregated proteins, invading pathogens, chemical insults, or other stressors, several outcomes are possible: (1) Immediate resealing of lysosomal membranes through stress granule-mediated repair. (2) Activation of multiple repair mechanisms, including ESCRTs dependent and independent mechanisms. (3) Mildly damaged lysosomes can be repaired, while severely damaged lysosomes are targeted for removal via autophagy (lysophagy). (4) Lysosomal damage also promotes lysosomal biogenesis through the activation of TFEB, regulated by mTORC1, calcium signaling, and other pathways. (5) In cases where repair is insufficient, damaged lysosomes may be expelled through fusion with the plasma membrane. The term *rejacere* (Latin: to expel) is used here to describe the active expulsion of lysosomal contents, emphasizing its mechanistic and evolutionary context. (6) If repair and removal mechanisms fail, the release of lysosomal contents such as cathepsins, coupled with altered signaling pathways at the lysosomal signaling hub, can trigger lysosomal cell death via multiple mechanisms.

The progression of lysosome-related diseases often correlates more closely with alternative strategies employed by lysosomes when conventional repair mechanisms fail, particularly in the contexts of age-related diseases and pathogen infections. This review aims to critically examine these alternative strategies, including different forms of lysosomal damage-related secretion, and the role ER and Golgi aparatus play in the repair and handling of damged lysosomes—other important aspects related to lysosomal damage repair, such as lysosomal repositioning and reformation, and lysosomal cell death (Figure 2) are reviewed elsewhere (Aits and Jäättelä, 2013; Gómez-Sintes et al., 2016; Pu et al., 2016; Scerra et al., 2022; Wang et al., 2018), therefore would not be covered here. By addressing these relatively neglected areas of lysosomal adaptation and alterations following lysosomal damage, this review seeks to provide a better understanding of the underlying mechanisms and their implications for cellular homeostasis and disease.

## Lysosomal damage and secretion

Under pathological conditions where lysosomes are severely damaged—such as during cancer treatment, pathogen infections, and neurodegenerative diseases—their repair capacities are often compromised or overwhelmed. In these scenarios, cells frequently adopt an alternative mechanism of expelling compromised lysosomes along with their enzymatic content and undigested materials (Chen et al., 2021; Domingues et al., 2024; Sho et al., 2024; Wang et al., 2023; Zhitomirsky and Assaraf, 2017; Zhong et al., 2023). These expelled materials may include lysosomotropic agents, engulfed pathogens, and aggregated pathogenic proteins. Furthermore, vesicles and organelles within the endolysosomal system that are primed to fuse with lysosomes—such as autophagosomes, endosomes, and MVBs—as well as damaged organelles engulfed by autophagosomes or endosomes [e.g., mitochondria (Bao et al., 2022; Liang et al., 2023)], may also be secreted concurrently or in parallel with lysosomal expulsion.

These lysosome-associated secretion processes can significantly influence disease susceptibility and progression (Groth-Pedersen and Jaattela, 2013; Scotto Rosato et al., 2022; Xu et al., 2021). For instance, the expulsion of aggregated proteins or pathogens through this mechanism has been linked to the spread of pathogenic agents in neurodegenerative diseases and the survival of invasive pathogens in infections. In cancer, lysosomal exocytosis contributes to drug resistance by releasing lysosomotropic chemotherapeutic agents from cells (Machado et al., 2015; Zhitomirsky and Assaraf, 2017). Conversely, under specific circumstances, lysosomal secretion may serve a protective role, such as by removing cytotoxic material or reducing intracellular stress (Tsunemi et al., 2019; Zhong et al., 2023).

The impact of lysosome-associated secretion on diseases underscores a potentially underappreciated relationship between this process and disease mechanisms. By better understanding the molecular pathways governing lysosomal secretion, therapeutic targeting of lysosome-associated secretion could represent a novel and promising strategy for managing diseases characterized by lysosomal dysfunction, including neurodegenerative disorders, infectious diseases, and cancers.

### Lysosomal exocytosis

Lysosomal exocytosis is a calcium-dependent process in which lysosomes fuse directly with the plasma membrane to release their contents into the extracellular space (Zhong et al., 2023). This mechanism is present in all cell types and is activated by a variety of stimuli. It plays a crucial role in numerous physiological processes, including plasma membrane repair (Reddy et al., 2001), bone resorption (Kim et al., 2024; Lee et al., 2024), pigmentation (Stinchcombe et al., 2004; Wünkhaus et al., 2024), immune responses (Brady et al., 2018; Jin et al., 2021; Sáez et al., 2019), mitosis (Hämälistö et al., 2020; Nugues et al., 2022), and ATP release in the nervous system (Jung et al., 2013; Shin et al., 2012; Zhang et al., 2007). Moreover, when lysosomes are damaged or their functions are compromised, the exocytosis process is typically enhanced (Bogacki et al., 2025; Chen et al., 2011; Domingues et al., 2024; Eriksson et al., 2020; Ghosh et al., 2020; Sivaramakrishnan et al., 2012; Wang et al., 2023). This enhancement is triggered by events such as lysosomal membrane permeabilization, alkalinization, and the consequent release of Ca^2+^, all of which can induce cellular stress and inflammation.

Enhanced lysosomal exocytosis can have both beneficial and detrimental effects, depending on the pathological context. For instance, in cancer treatment, the release of lysosomal enzymes through lysosomal exocytosis is often associated with increased metastasis and reduced patient survival (Machado et al., 2015; Ren et al., 2025). Conversely, the expulsion of lysosomotropic agents via lysosomal exocytosis in renal proximal tubular epithelial cells—mediated by the activation of the lysosomal Ca^2+^ channel TRPML1—has been shown to mitigate uranium-induced nephrotoxicity (Zhong et al., 2023). This dichotomy underscores the critical influence of the specific components released during lysosomal exocytosis.

In neurodegenerative diseases, the accumulation of aggregated misfolded proteins is a central factor in disease progression. The role of lysosomes in degrading these proteins—or alternatively, in their activation, accumulation, or release—is paramount. For example, in synucleinopathy models, lysosomal exocytosis-mediated release of degradation-resistant α-synuclein species from neurons has been identified as a key mechanism for the propagation of pathogenic α-synuclein in mouse brains (Xie et al., 2022). However, contrasting findings indicate that activation of TRPML1 can protect human dopaminergic neurons by rescuing defective α-synuclein secretion and preventing its accumulation (Tsunemi et al., 2019). This again suggests that the effects of lysosomal exocytosis in neurodegenerative diseases may vary depending on the specific cellular context involved.

Similarly, during pathogen infections, lysosomal damage-induced exocytosis serves as an effective mechanism to expel overwhelmed pathogens (Chen et al., 2021; Deretic and Wang, 2023; Ghosh et al., 2020; Koo et al., 2008; Shtuhin-Rahav et al., 2023; Wang et al., 2023). However, this process can be hijacked by pathogens to facilitate their own survival and dissemination. For example, both severe acute respiratory syndrome coronavirus 2 (SARS-CoV-2) (Chen et al., 2021) and its β-coronavirus relative MHV (Ghosh et al., 2020) induce lysosomal damage and exocytosis through multiple Ca^2+^-dependent mechanisms, enabling their egress from infected cells. Additionally, in bacterial infections, lysosomal exocytosis from immune and other cell types is associated with the cytolytic effects of bacteria, allowing bacteria within lysosomes and phagosomes to evade digestion and enhance their survival (Koo et al., 2008; Shtuhin-Rahav et al., 2023; Wang et al., 2023).

These studies illustrate that lysosomal exocytosis plays a complex role in disease dynamics, acting as a double-edged sword. On one hand, it can protect cells by removing toxic substances and pathogens; on the other hand, it can facilitate disease progression by promoting metastasis, protein propagation, and pathogen survival. Understanding the specific context of lysosomal exocytosis is therefore crucial.

### Alternative secretion mechanisms

Besides lysosomal exocytosis, at least two additional secretion mechanisms are linked to lysosomal damage and compromised lysosomal function: exosome secretion and secretory autophagy. Exosome secretion is a process in which small extracellular vesicles, known as exosomes, are released following the fusion of MVBs with the plasma membrane (Han et al., 2022). The formation of exosomes is facilitated by the ESCRT complexes, which sort cargo into intraluminal vesicles and drive membrane inward budding events. These exosomes, which transport proteins, lipids, and nucleic acids, play a crucial role in intercellular communication and modulate various physiological and pathological processes. Secretory autophagy, on the other hand, repurposes the cellular autophagic machinery to facilitate the secretion of cellular components, rather than their degradation (Buratta et al., 2020; Debnath and Leidal, 2022; Solvik et al., 2022). Unlike the classical autophagic pathway, which typically directs cargo to lysosomes for degradation, secretory autophagy allows the release of cellular materials, such as proteins and lipids, into the extracellular space. This process is particularly important for cellular communication, immune responses, and tissue homeostasis, providing an alternative route for the secretion of factors like cytokines and extracellular matrix components. Both exosome secretion and secretory autophagy serve as alternative pathways for managing cargo within the endolysosomal system, contributing to cellular responses and disease progression by facilitating the extracellular release of materials that would otherwise be degraded.

A significant feature of approximately half of the lysosomal storage disorders (LSDs)—a group of over 70 rare inherited metabolic diseases caused by lysosomal dysfunction—is the increased secretion of exosomes, highlighting the correlation between lysosomal impairment and exosome release (Abe et al., 2024; Alvarez-Erviti et al., 2011). The exosome-mediated release of pathogenic α-synuclein from macrophage lineage cells or neuroblastoma cells has been attributed to lysosomal stress-induced dysfunction (Abe et al., 2024; Alvarez-Erviti et al., 2011). In another model of lysosomal dysfunction, the contents released via exosomes include amyloid precursor protein C-terminal fragments (APP-CTFs), specific sphingolipids, and the phospholipid bis(monoacylglycero)phosphate (BMP), which normally reside in the internal vesicles of endolysosomes (Miranda et al., 2018). Notably, disruption of endolysosome fusion also increases exosome secretion (Shelke et al., 2023), indicating that the physical interaction between lysosomes and MVBs inherently predicts exosome release.

However, when lysosomal inhibition is induced by agents such as chloroquine or Bafilomycin A, cells tend to utilize secretory autophagy instead of exosome secretion, as evidenced by increased secretion of autophagy cargo receptors in extracellular vesicles and particles (EVPs) but negligible changes in classical exosome markers such as TSG101, ALIX, and CD9 (Solvik et al., 2022). This observation is consistent with several other studies demonstrating that lysosomal damage or stress can induce secretory autophagy (Chang et al., 2024; Dash et al., 2024; Kimura et al., 2017). Considering the significant roles of secretory autophagy and exosome secretion in the progression of diseases such as neurodegenerative disorders and cancer, as well as the use of exosomes for drug delivery, the interplay between lysosomal damage and secretion warrants further attention.

## Lysosomal damage and endomembrane system

While much attention has been given to the endolysosomal system itself, emerging research reveals that lysosomes rely heavily on interactions with other organelles—particularly the endoplasmic reticulum (ER) and Golgi apparatus—to repair damage and restore homeostasis.

### ER and lysosomal damage

Emerging evidence underscores the critical role of endoplasmic reticulum (ER)-lysosome membrane contact sites (MCSs) in mediating lysosomal repair. Central to this process is the phosphatidylinositol-4 kinase type 2α (PI4K2A), which generates phosphatidylinositol-4-phosphate (PI4P) at lysosomal membranes. PI4P recruits oxysterol-binding protein (OSBP)-related protein (ORP) family members, including ORP9, ORP10, ORP11, and OSBP, to orchestrate the formation of ER-lysosome MCSs (Tan and Finkel, 2022). These dynamic contact sites facilitate the transfer of phosphatidylserine and cholesterol from the ER to damaged lysosomes, enabling rapid membrane restoration. Complementary studies emphasize the role of cholesterol and ER-resident tethering proteins, such as VAPA/B and ORP1L, in stabilizing these interactions and promoting lysosomal integrity (Radulovic et al., 2022).

A parallel mechanism involves the lipid transfer protein ATG2, which is recruited to damaged lysosomes to mediate direct lipid transfer for membrane repair (Tan and Finkel, 2022). ATG2’s interaction with the lipid scramblase ATG9, essential for ER-phagophore contact site formation and autophagosome maturation (Gomez-Sanchez et al., 2018; van Vliet et al., 2022), suggests a coordinated interplay between lysosomal repair and lysophagy. This duality highlights a potential regulatory axis where ER-driven lipid redistribution supports both autophagosome biogenesis and lysosomal membrane restoration.

Pathophysiological insights emerge from studies linking ER-lysosome interactions to Parkinson’s disease (PD). VPS13C and LRRK2, two PD-associated proteins, are recruited to damaged lysosomes: VPS13C facilitates ER-lysosome tethering, while LRRK2 promotes lysosomal membrane tubulation and cargo sorting (Bonet-Ponce and Cookson, 2022; Wang et al., 2024). Similarly, PDZD8, a tubular lipid-binding protein (TULIP) superfamily member, tethers ER-lysosome MCSs to regulate lysosome maturation and autophagy (Guillén-Samander et al., 2019; Thakur and O’Connor-Giles, 2023). Behavioral abnormalities observed in PDZD8-deficient mice (Kurihara et al., 2023) raise intriguing questions about whether these phenotypes come partially from disrupted ER-lysosome communication, underscoring the need to explore MCS dysfunction in neurodegenerative contexts.

The endoplasmic reticulum (ER) may contribute to lysosomal damage repair through additional mechanisms. For example, during lysosomal injury, calcium efflux from damaged lysosomes has been shown to trigger compensatory ER-mediated calcium refilling in multiple models of lysosomal dysfunction (Garrity et al., 2016; Kang et al., 2024; Liu and Lieberman, 2019). Given the central role of calcium signaling in lysosomal repair and adaptation, future studies should investigate whether ER-lysosome membrane contact sites (MCSs) directly facilitate this calcium replenishment process. Such work could reveal how spatial and temporal coordination between calcium homeostasis and lipid transfer synergistically enhances lysosomal membrane repair. Beyond calcium dynamics, the ER and ER-Golgi intermediate compartment (ERGIC) also play critical roles in autophagy, a process tightly linked to lysosomal recovery. The ER and ERGIC are well-known sources of membranes for autophagosome biogenesis, supplying lipids and proteins required for phagophore expansion (Han et al., 2023; Melia et al., 2020). Building on these findings, the interplay between ER/ERGIC-driven autophagosome formation and lysosomal repair mechanisms—such as lysophagy—remains an open question. For instance, it is unclear whether ER-derived autophagosomes are preferentially recruited to engulf damaged lysosomes or if their maturation is synchronized with lysophagy. Elucidating these interactions could clarify how membrane trafficking pathways converge to restore lysosomal function, offering insights into the integration of autophagy and organelle repair. By exploring these mechanisms, researchers could refine models of lysosomal recovery, emphasizing the ER’s dual role in calcium regulation and membrane supply, and how these functions are coordinated to resolve lysosomal stress.

### Golgi apparatus and lysosomal damage

In contrast to the ER and ERGIC, which directly interact with lysosomes through membrane contact sites to repair damage, the Golgi apparatus engages with lysosomes in a more indirect and unidirectional manner. This interaction primarily occurs via two pathways: 1) Protein Transport: Proteins, including hydrolases processed by the Golgi apparatus, are transported to endosomes through vesicular trafficking. These proteins ultimately reach lysosomes through acidification and fusion within the endolysosomal pathway (Melia et al., 2020; Scheuring et al., 2011). Therefore, deficiencies in posttranslational modifications within the Golgi apparatus are strongly associated with lysosomal dysfunction-related diseases (Akaaboune and Wang, 2024; Sou et al., 2024). 2) Autophagosome Biogenesis: The Golgi apparatus also serves as a source of key proteins and membrane components necessary for autophagosome formation (Gao et al., 2016; Sawa-Makarska et al., 2020). These components are incorporated into autophagosomes, which subsequently fuse with lysosomes to form autolysosomes, thereby delivering their cargo for degradation.

Despite these established pathways, the interactions between the Golgi apparatus and lysosomes might be more complex. In numerous diseases characterized by lysosomal dysfunction—such as lysosomal storage disorders (Lojewski et al., 2014; Shammas et al., 2019), neurodegenerative diseases (Gosavi et al., 2002; Martínez-Menárguez et al., 2019), COVID-19 infection (Cortese et al., 2020; Zhang et al., 2024), nicotine exposure (Govind et al., 2021), and epilepsy or other mental disorders triggered by electrical signal disturbances (Thayer et al., 2013)—morphological alterations in the Golgi apparatus have been consistently observed. These alterations include fragmentation (reduction in Golgi stack organization and increased dispersal), vesiculation (increased formation of Golgi-derived vesicles), and depolarization (randomized Golgi distribution), indicating that lysosomal damage and Golgi apparatus disorganization are concomitant events in these pathologies.

Similar to the ER, several Golgi-resident proteins may be recruited to or localized near damaged lysosomes, further suggesting a more complex interplay between the Golgi apparatus and lysosomal dysfunction. For example, Rab34, a Golgi-localized protein, plays a significant role in lysosome positioning and function. Overexpression or constitutive activation of Rab34 relocates lysosomes to the peri-Golgi area (Kumar et al., 2024; Wang and Hong, 2002) and facilitates the fusion of phagosomes with lysosomes (Seto et al., 2011). Additionally, loss-of-function variants of Rab34 are associated with various ciliopathies (Batkovskyte et al., 2024; Bruel et al., 2023), suggesting the possibility that dysregulation of Rab34-mediated Golgi-lysosome interactions may contribute to the pathogenesis of human diseases. The possibility of Golgi–lysosome interaction is further supported by single-organelle immunoprecipitation–coupled mass spectrometry studies of the Golgi (Fasimoye et al., 2023) and lysosomes (Eapen et al., 2021; Jia et al., 2022; Tan and Finkel, 2022; Wyant et al., 2018), underscoring the need for continued investigation.

## Discussion

Lysosomal quality control is vital for cellular homeostasis and disease progression, with lysosomes employing alternative secretion pathways, such as exocytosis, exosome release, and secretory autophagy, when traditional repair mechanisms fail. Interactions with the endomembrane system, particularly the ER and Golgi apparatus, are also essential for lysosomal repair and function. These pathways play key roles in diseases, such as neurodegenerative disorders, where exosome-mediated spread of aggregated proteins accelerates progression, and cancer, where lysosomal exocytosis contributes to drug resistance and metastasis. Pathogens also exploit lysosomal secretion to enhance survival and spread, highlighting its complex role in disease.

Targeting lysosomal secretion and enhancing organelle interactions offer promising treatment strategies for lysosome-related diseases. Modulating exosome release could limit pathogenic protein spread in neurodegeneration, while inhibiting lysosomal exocytosis may help overcome cancer drug resistance. Strengthening ER-lysosome and Golgi-lysosome interactions could enhance lysosomal resilience in various diseases. These approaches aim to mitigate lysosomal dysfunction and improve cellular stress responses, offering new therapeutic perspectives.

Despite progress, key questions remain in lysosomal biology. Future research should focus on how lysosomal positioning impacts quality control, identifying specialized lysosome subpopulations, and understanding coordination with organelles like mitochondria. Additionally, the molecular triggers behind Golgi morphological changes in response to lysosomal damage and their role in repair need further investigation. Addressing these questions is critical to fully understanding lysosomal function and its integration within the cellular network.

In conclusion, lysosomes employ diverse strategies, including alternative secretion pathways and organelle interactions, to maintain cellular homeostasis under stress. These mechanisms are crucial in diseases like neurodegeneration, cancer, and infections. A deeper understanding of lysosomal-endomembrane interactions will uncover new therapeutic targets and help improve strategies for managing diseases linked to lysosomal dysfunction.

## References

[B1] Abe T. Kuwahara Suenaga T. Sakurai S. Takatori M. S. Iwatsubo T. (2024). Lysosomal stress drives the release of pathogenic α-synuclein from macrophage lineage cells via the LRRK2-Rab10 pathway. iScience 27, 108893. 10.1016/j.isci.2024.108893 38313055 PMC10835446

[B2] Aits S. Jäättelä M. (2013). Lysosomal cell death at a glance. J. Cell Sci. 126, 1905–1912. 10.1242/jcs.091181 23720375

[B3] Akaaboune S. R. Wang Y. (2024). Golgi defect as a major contributor to lysosomal dysfunction. Front. Cell Dev. Biol. 12, 1386149. 10.3389/fcell.2024.1386149 38721528 PMC11076776

[B4] Almeida M. F. Bahr B. A. Kinsey S. T. (2020). Endosomal-lysosomal dysfunction in metabolic diseases and Alzheimer's disease. Int. Rev. Neurobiol. 154, 303–324. 10.1016/bs.irn.2020.02.012 32739009 PMC8428780

[B5] Alvarez-Erviti L. Seow Y. Schapira A. H. Gardiner C. Sargent I. L. Wood M. J. (2011). Lysosomal dysfunction increases exosome-mediated alpha-synuclein release and transmission. Neurobiol. Dis. 42, 360–367. 10.1016/j.nbd.2011.01.029 21303699 PMC3107939

[B6] Bakowski M. A. Braun V. Lam G. Y. Yeung T. Heo W. D. Meyer T. (2010). The phosphoinositide phosphatase SopB manipulates membrane surface charge and trafficking of the Salmonella-containing vacuole. Cell Host Microbe 7, 453–462. 10.1016/j.chom.2010.05.011 20542249

[B7] Ballabio A. Bonifacino J. S. (2020). Lysosomes as dynamic regulators of cell and organismal homeostasis. Nat. Rev. Mol. Cell Biol. 21, 101–118. 10.1038/s41580-019-0185-4 31768005

[B8] Bao F. Zhou L. Zhou R. Huang Q. Chen J. Zeng S. (2022). Mitolysosome exocytosis, a mitophagy-independent mitochondrial quality control in flunarizine-induced parkinsonism-like symptoms. Sci. Adv. 8, eabk2376. 10.1126/sciadv.abk2376 35417232 PMC9007515

[B9] Batkovskyte D. Komatsu M. Hammarsjö A. Pooh R. Shimokawa O. Ikegawa S. (2024). Compound heterozygous variants in RAB34 in a rare skeletal ciliopathy syndrome. Clin. Genet. 105, 87–91. 10.1111/cge.14419 37619988

[B11] Bhattacharya A. Mukherjee R. Kuncha S. K. Brunstein M. E. Rathore R. Junek S. (2023). A lysosome membrane regeneration pathway depends on TBC1D15 and autophagic lysosomal reformation proteins. Nat. Cell Biol. 25, 685–698. 10.1038/s41556-023-01125-9 37024685

[B12] Bogacki E. C. Longmore G. Lewis P. A. Herbst S. (2025). GPNMB is a biomarker for lysosomal dysfunction and is secreted via LRRK2-modulated lysosomal exocytosis. bioRxiv, 2001–630988. 10.1101/2025.01.01.630988 PMC13142706 41406229

[B13] Bonet-Ponce L. Cookson M. R. (2022). The endoplasmic reticulum contributes to lysosomal tubulation/sorting driven by LRRK2. Mol. Biol. Cell 33, ar124. 10.1091/mbc.E22-04-0139 36044336 PMC9634967

[B14] Brady O. A. Martina J. A. Puertollano R. (2018). Emerging roles for TFEB in the immune response and inflammation. Autophagy 14, 181–189. 10.1080/15548627.2017.1313943 28738171 PMC5902167

[B15] Bruel A. L. Ganga A. K. Nosková L. Valenzuela I. Martinovic J. Duffourd Y. (2023). Pathogenic RAB34 variants impair primary cilium assembly and cause a novel oral-facial-digital syndrome. Hum. Mol. Genet. 32, 2822–2831. 10.1093/hmg/ddad109 37384395 PMC10481091

[B16] Buratta S. Tancini B. Sagini K. Delo F. Chiaradia E. Urbanelli L. (2020). Lysosomal exocytosis, exosome release and secretory autophagy: the autophagic- and endo-lysosomal systems go extracellular. Int. J. Mol. Sci. 21, 2576. 10.3390/ijms21072576 32276321 PMC7178086

[B17] Bussi C. Mangiarotti A. Vanhille-Campos C. Aylan B. Pellegrino E. Athanasiadi N. (2023). Stress granules plug and stabilize damaged endolysosomal membranes. Nature 623, 1062–1069. 10.1038/s41586-023-06726-w 37968398 PMC10686833

[B18] Chang Y.-C. Gao Y. Lee J. Y. Peng Y.-J. Langen J. Chang K. T. (2024). Identification of secretory autophagy as a mechanism modulating activity-induced synaptic remodeling, Proc. Natl. Acad. Sci. U. S. A., 121, e2315958121, 10.1073/pnas.2315958121 38588427 PMC11032469

[B19] Chen D. Zheng Q. Sun L. Ji M. Li Y. Deng H. (2021). ORF3a of SARS-CoV-2 promotes lysosomal exocytosis-mediated viral egress. Dev. Cell 56, 3250–3263.e5. 10.1016/j.devcel.2021.10.006 34706264 PMC8502680

[B20] Chen P. M. Gombart Z. J. Chen J. W. (2011). Chloroquine treatment of ARPE-19 cells leads to lysosome dilation and intracellular lipid accumulation: possible implications of lysosomal dysfunction in macular degeneration. Cell and Biosci. 1, 10. 10.1186/2045-3701-1-10 PMC3125200 21711726

[B21] Cortese M. Lee J. Y. Cerikan B. Neufeldt C. J. Oorschot V. M. J. Kohrer S. (2020). Integrative imaging reveals SARS-CoV-2-induced reshaping of subcellular morphologies. Cell Host Microbe 28, 853–866. 10.1016/j.chom.2020.11.003 33245857 PMC7670925

[B22] Dash B. K. Urano Y. Noguchi N. (2024). Lysosomal damage promotes autophagy-based unconventional secretion of the Parkinson’s disease protein PARK7. Redox Exp. Med. 2024 2024. 10.1530/rem-24-0014

[B23] Debnath J. Leidal A. M. (2022). Secretory autophagy during lysosome inhibition (SALI). Autophagy 18, 2498–2499. 10.1080/15548627.2022.2095788 35786367 PMC9542525

[B24] Deretic V. Wang F. (2023). Autophagy is part of the answer to tuberculosis. Nat. Microbiol. 8, 762–763. 10.1038/s41564-023-01373-3 37142685 PMC10636698

[B25] De Tito S. Hervás J. H. van Vliet A. R. Tooze S. A. (2020). The Golgi as an assembly line to the autophagosome. Trends Biochem. Sci. 45, 484–496. 10.1016/j.tibs.2020.03.010 32307224

[B26] Domingues N. Catarino S. Cristóvão B. Rodrigues L. Carvalho F. A. Sarmento M. J. (2024). Connexin43 promotes exocytosis of damaged lysosomes through actin remodelling. Embo J. 43, 3627–3649. 10.1038/s44318-024-00177-3 39044100 PMC11377567

[B27] Duran J. Salinas J. E. Wheaton R. P. Poolsup S. Allers L. Rosas-Lemus M. (2024). Calcium signaling from damaged lysosomes induces cytoprotective stress granules. EMBO J. 43, 6410–6443. 10.1038/s44318-024-00292-1 39533058 PMC11649789

[B28] Eapen V. V. Swarup S. Hoyer M. J. Paulo J. A. Harper J. W. (2021). Quantitative proteomics reveals the selectivity of ubiquitin-binding autophagy receptors in the turnover of damaged lysosomes by lysophagy. Elife 10, e72328. 10.7554/eLife.72328 34585663 PMC8523161

[B29] Ebstrup M. L. Sonder S. L. Fogde D. L. Heitmann A. S. B. Dietrich T. N. Dias C. (2023). Annexin A7 mediates lysosome repair independently of ESCRT-III. Front. Cell Dev. Biol. 11, 1211498. 10.3389/fcell.2023.1211498 38348092 PMC10860759

[B30] Eriksson I. Waster P. Ollinger K. (2020). Restoration of lysosomal function after damage is accompanied by recycling of lysosomal membrane proteins. Cell Death Dis. 11, 370. 10.1038/s41419-020-2527-8 32409651 PMC7224388

[B31] Fasimoye R. Dong W. Nirujogi R. S. Rawat E. S. Iguchi M. Nyame K. (2023). Golgi-IP, a tool for multimodal analysis of Golgi molecular content, Proc. Natl. Acad. Sci. U. S. A., 120, e2219953120, 10.1073/pnas.2219953120 37155866 PMC10193996

[B32] Gahlot P. Kravic B. Rota G. van den Boom J. Levantovsky S. Schulze N. (2024). Lysosomal damage sensing and lysophagy initiation by SPG20-ITCH. Mol. Cell 84, 1556–1569.e10. 10.1016/j.molcel.2024.02.029 38503285

[B33] Gao Y. Liu Y. Hong L. Yang Z. Cai X. Chen X. (2016). Golgi-associated LC3 lipidation requires V-ATPase in noncanonical autophagy. Cell Death and Dis. 7, e2330. 10.1038/cddis.2016.236 PMC5108321 27512951

[B34] Garrity A. G. Wang W. Collier C. M. Levey S. A. Gao Q. Xu H. (2016). The endoplasmic reticulum, not the pH gradient, drives calcium refilling of lysosomes. Elife 5, e15887. 10.7554/eLife.15887 27213518 PMC4909396

[B36] Ghosh S. Dellibovi-Ragheb T. A. Kerviel A. Pak E. Qiu Q. Fisher M. (2020). β-Coronaviruses use lysosomes for egress instead of the biosynthetic secretory pathway. Cell 183, 1520–1535. 10.1016/j.cell.2020.10.039 33157038 PMC7590812

[B37] Gomez-Sanchez R. Rose J. Guimaraes R. Mari M. Papinski D. Rieter E. (2018). Atg9 establishes Atg2-dependent contact sites between the endoplasmic reticulum and phagophores. J. Cell Biol. 217, 2743–2763. 10.1083/jcb.201710116 29848619 PMC6080931

[B38] Gómez-Sintes R. Ledesma M. D. Boya P. (2016). Lysosomal cell death mechanisms in aging. Ageing Res. Rev. 32, 150–168. 10.1016/j.arr.2016.02.009 26947122

[B39] Gosavi N. Lee H. J. Lee J. S. Patel S. Lee S. J. (2002). Golgi fragmentation occurs in the cells with prefibrillar alpha-synuclein aggregates and precedes the formation of fibrillar inclusion. J. Biol. Chem. 277, 48984–48992. 10.1074/jbc.M208194200 12351643

[B40] Govind A. P. Jeyifous O. Russell T. A. Yi Z. Weigel A. V. Ramaprasad A. (2021). Activity-dependent Golgi satellite formation in dendrites reshapes the neuronal surface glycoproteome. Elife 10, e68910. 10.7554/eLife.68910 34545811 PMC8494481

[B41] Groth-Pedersen L. Jaattela M. (2013). Combating apoptosis and multidrug resistant cancers by targeting lysosomes. Cancer Lett. 332, 265–274. 10.1016/j.canlet.2010.05.021 20598437

[B42] Guillén-Samander A. Bian X. De Camilli P. (2019). PDZD8 mediates a Rab7-dependent interaction of the ER with late endosomes and lysosomes. Proc. Natl. Acad. Sci. 116, 22619–22623. 10.1073/pnas.1913509116 31636202 PMC6842579

[B43] Hämälistö S. Stahl J. L. Favaro E. Yang Q. Liu B. Christoffersen L. (2020). Spatially and temporally defined lysosomal leakage facilitates mitotic chromosome segregation. Nat. Commun. 11, 229. 10.1038/s41467-019-14009-0 31932607 PMC6957743

[B44] Han Q. F. Li W. J. Hu K. S. Gao J. Zhai W. L. Yang J. H. (2022). Exosome biogenesis: machinery, regulation, and therapeutic implications in cancer. Mol. Cancer 21, 207. 10.1186/s12943-022-01671-0 36320056 PMC9623991

[B45] Han Y. Li S. Ge L. (2023). Biogenesis of autophagosomes from the ERGIC membrane system. J. Genet. Genomics 50, 3–6. 10.1016/j.jgg.2022.07.001 35835319

[B46] Hu Y.-B. Dammer E. B. Ren R.-J. Wang G. (2015). The endosomal-lysosomal system: from acidification and cargo sorting to neurodegeneration. Transl. Neurodegener. 4, 18. 10.1186/s40035-015-0041-1 26448863 PMC4596472

[B47] Jia J. Abudu Y. P. Claude-Taupin A. Gu Y. Kumar S. Choi S. W. (2018). Galectins control mTOR in response to endomembrane damage. Mol. Cell 70, 120–135. 10.1016/j.molcel.2018.03.009 29625033 PMC5911935

[B48] Jia J. Abudu Y. P. Claude-Taupin A. Gu Y. Kumar S. Choi S. W. (2019). Galectins control MTOR and AMPK in response to lysosomal damage to induce autophagy. Autophagy 15, 169–171. 10.1080/15548627.2018.1505155 30081722 PMC6287685

[B49] Jia J. Bissa B. Brecht L. Allers L. Choi S. W. Gu Y. (2020a). AMPK, a regulator of metabolism and autophagy, is activated by lysosomal damage via a novel galectin-directed ubiquitin signal transduction system. Mol. Cell 77, 951–969. 10.1016/j.molcel.2019.12.028 31995728 PMC7785494

[B50] Jia J. Claude-Taupin A. Gu Y. Choi S. W. Peters R. Bissa B. (2020b). Galectin-3 coordinates a cellular system for lysosomal repair and removal. Dev. Cell 52, 69–87. 10.1016/j.devcel.2019.10.025 31813797 PMC6997950

[B51] Jia J. Wang F. Bhujabal Z. Peters R. Mudd M. Duque T. (2022). Stress granules and mTOR are regulated by membrane atg8ylation during lysosomal damage. J. Cell Biol. 221, e202207091. 10.1083/jcb.202207091 36179369 PMC9533235

[B52] Jin J. Zhang H. Weyand C. M. Goronzy J. J. (2021). Lysosomes in T Cell immunity and aging. Front. Aging 2, 809539. 10.3389/fragi.2021.809539 35822050 PMC9261317

[B53] Jung J. Shin Y. H. Konishi H. Lee S. J. Kiyama H. (2013). Possible ATP release through lysosomal exocytosis from primary sensory neurons. Biochem. Biophysical Res. Commun. 430, 488–493. 10.1016/j.bbrc.2012.12.009 23237805

[B54] Kang H. Choi S. W. Kim J. Y. Oh S.-J. Kim S. J. Lee M.-S. (2024). ER-to-lysosome Ca2+ refilling followed by K+ efflux-coupled store-operated Ca2+ entry in inflammasome activation and metabolic inflammation. eLife Sci. Publ. Ltd. 12. 10.7554/elife.87561.3 PMC11219040 38953285

[B55] Kim M. Park J. H. Go M. Lee N. Seo J. Lee H. (2024). RUFY4 deletion prevents pathological bone loss by blocking endo-lysosomal trafficking of osteoclasts. Bone Res. 12, 29. 10.1038/s41413-024-00326-8 38744829 PMC11094054

[B56] Kimura T. Jia J. Kumar S. Choi S. W. Gu Y. Mudd M. (2017). Dedicated SNAREs and specialized TRIM cargo receptors mediate secretory autophagy. EMBO J. 36, 42–60. 10.15252/embj.201695081 27932448 PMC5210154

[B57] Koo I. C. Wang C. Raghavan S. Morisaki J. H. Cox J. S. Brown E. J. (2008). ESX-1-dependent cytolysis in lysosome secretion and inflammasome activation during mycobacterial infection. Cell Microbiol. 10, 1866–1878. 10.1111/j.1462-5822.2008.01177.x 18503637 PMC2574867

[B58] Kumar R. Khan M. Francis V. Aguila A. Kulasekaran G. Banks E. (2024). DENND6A links Arl8b to a Rab34/RILP/dynein complex, regulating lysosomal positioning and autophagy. Nat. Commun. 15, 919. 10.1038/s41467-024-44957-1 38296963 PMC10830484

[B59] Kurihara Y. Mitsunari K. Mukae N. Shoji H. Miyakawa T. Shirane M. (2023). PDZD8-deficient mice manifest behavioral abnormalities related to emotion, cognition, and adaptation due to dyslipidemia in the brain. Mol. Brain 16, 11. 10.1186/s13041-023-01002-4 36658656 PMC9854033

[B60] Lee J. J. Wang T. Wiggins K. Lu P. N. Underwood C. Ochenkowska K. (2024). Dysregulated lysosomal exocytosis drives protease-mediated cartilage pathogenesis in multiple lysosomal disorders. iScience 27, 109293. 10.1016/j.isci.2024.109293 38495824 PMC10940929

[B61] Liang W. Sagar S. Ravindran R. Najor R. H. Quiles J. M. Chi L. (2023). Mitochondria are secreted in extracellular vesicles when lysosomal function is impaired. Nat. Commun. 14, 5031. 10.1038/s41467-023-40680-5 37596294 PMC10439183

[B62] Lie P. P. Y. Yang D. S. Stavrides P. Goulbourne C. N. Zheng P. Mohan P. S. (2021). Post-Golgi carriers, not lysosomes, confer lysosomal properties to pre-degradative organelles in normal and dystrophic axons. Cell Rep. 35, 109034. 10.1016/j.celrep.2021.109034 33910020 PMC8135226

[B63] Liu E. A. Lieberman A. P. (2019). The intersection of lysosomal and endoplasmic reticulum calcium with autophagy defects in lysosomal diseases. Neurosci. Lett. 697, 10–16. 10.1016/j.neulet.2018.04.049 29704574 PMC6202281

[B64] Lojewski X. Staropoli J. F. Biswas-Legrand S. Simas A. M. Haliw L. Selig M. K. (2014). Human iPSC models of neuronal ceroid lipofuscinosis capture distinct effects of TPP1 and CLN3 mutations on the endocytic pathway. Hum. Mol. Genet. 23, 2005–2022. 10.1093/hmg/ddt596 24271013 PMC3959814

[B65] Machado E. White-Gilbertson S. van de Vlekkert D. Janke L. Moshiach S. Campos Y. (2015). Regulated lysosomal exocytosis mediates cancer progression. Sci. Adv. 1, e1500603. 10.1126/sciadv.1500603 26824057 PMC4730843

[B66] Maejima I. Takahashi A. Omori H. Kimura T. Takabatake Y. Saitoh T. (2013). Autophagy sequesters damaged lysosomes to control lysosomal biogenesis and kidney injury. EMBO J. 32, 2336–2347. 10.1038/emboj.2013.171 23921551 PMC3770333

[B67] Martínez-Menárguez J. Tomás M. Martínez-Martínez N. Martínez-Alonso E. (2019). Golgi fragmentation in neurodegenerative diseases: is there a common cause? Cells 8, 748. 10.3390/cells8070748 31331075 PMC6679019

[B68] Melia T. J. Lystad A. H. Simonsen A. (2020). Autophagosome biogenesis: from membrane growth to closure. J. Cell Biol. 219, e202002085. 10.1083/jcb.202002085 32357219 PMC7265318

[B69] Miranda A. M. Lasiecka Z. M. Xu Y. Neufeld J. Shahriar S. Simoes S. (2018). Neuronal lysosomal dysfunction releases exosomes harboring APP C-terminal fragments and unique lipid signatures. Nat. Commun. 9, 291. 10.1038/s41467-017-02533-w 29348617 PMC5773483

[B70] Nascimbeni A. C. Giordano F. Dupont N. Grasso D. Vaccaro M. I. Codogno P. (2017). ER-plasma membrane contact sites contribute to autophagosome biogenesis by regulation of local PI3P synthesis. EMBO J. 36, 2018–2033. 10.15252/embj.201797006 28550152 PMC5509996

[B71] Neel E. Chiritoiu-Butnaru M. Fargues W. Denus M. Colladant M. Filaquier A. (2024). The endolysosomal system in conventional and unconventional protein secretion. J. Cell Biol. 223, e202404152. 10.1083/jcb.202404152 39133205 PMC11318669

[B72] Nugues C. Rajamanoharan D. Burgoyne R. D. Haynes L. P. Helassa N. (2022). Lysosome exocytosis is required for mitosis in mammalian cells. Biochem. Biophys. Res. Commun. 626, 211–219. 10.1016/j.bbrc.2022.08.024 35998546

[B73] Pan C. Banerjee K. Lehmann G. L. Almeida D. Hajjar K. A. Benedicto I. (2021). Lipofuscin causes atypical necroptosis through lysosomal membrane permeabilization, Proc. Natl. Acad. Sci. U. S. A., 118, e2100122118, 10.1073/pnas.2100122118 34782457 PMC8617501

[B74] Parenti G. Medina D. L. Ballabio A. (2021). The rapidly evolving view of lysosomal storage diseases. EMBO Mol. Med. 13, e12836. 10.15252/emmm.202012836 33459519 PMC7863408

[B75] Ponpuak M. Mandell M. A. Kimura T. Chauhan S. Cleyrat C. Deretic V. (2015). Secretory autophagy. Curr. Opin. Cell Biol. 35, 106–116. 10.1016/j.ceb.2015.04.016 25988755 PMC4529791

[B76] Pu J. Guardia C. M. Keren-Kaplan T. Bonifacino J. S. (2016). Mechanisms and functions of lysosome positioning. J. Cell Sci. 129, 4329–4339. 10.1242/jcs.196287 27799357 PMC5201012

[B77] Radulovic M. Schink K. O. Wenzel E. M. Nahse V. Bongiovanni A. Lafont F. (2018). ESCRT-mediated lysosome repair precedes lysophagy and promotes cell survival. EMBO J. 37, e99753. 10.15252/embj.201899753 30314966 PMC6213280

[B78] Radulovic M. Wenzel E. M. Gilani S. Holland L. K. Lystad A. H. Phuyal S. (2022). Cholesterol transfer via endoplasmic reticulum contacts mediates lysosome damage repair. EMBO J. 41, e112677. 10.15252/embj.2022112677 36408828 PMC9753466

[B79] Ramon-Luing L. A. Palacios Y. Ruiz A. Téllez-Navarrete N. A. Chavez-Galan L. (2023). Virulence factors of *Mycobacterium tuberculosis* as modulators of cell death mechanisms. Pathogens 12, 839. 10.3390/pathogens12060839 37375529 PMC10304248

[B80] Reddy A. Caler E. V. Andrews N. W. (2001). Plasma membrane repair is mediated by Ca(2+)-regulated exocytosis of lysosomes. Cell 106, 157–169. 10.1016/s0092-8674(01)00421-4 11511344

[B81] Ren W.-W. Kawahara R. Suzuki K. G. N. Dipta P. Yang G. Thaysen-Andersen M. (2025). MYO18B promotes lysosomal exocytosis by facilitating focal adhesion maturation. J. Cell Biol. 224, e202407068. 10.1083/jcb.202407068 39751400 PMC11697975

[B82] Richards C. M. Jabs S. Qiao W. Varanese L. D. Schweizer M. Mosen P. R. (2022). The human disease gene LYSET is essential for lysosomal enzyme transport and viral infection. Science 378, eabn5648. 10.1126/science.abn5648 36074821 PMC9547973

[B83] Rodgers S. J. Jones E. I. Arumugam S. Hamila S. A. Danne J. Gurung R. (2022). Endosome maturation links PI3Kα signaling to lysosome repopulation during basal autophagy. EMBO J. 41, e110398. 10.15252/embj.2021110398 35968799 PMC9531306

[B84] Root J. Merino P. Nuckols A. Johnson M. Kukar T. (2021). Lysosome dysfunction as a cause of neurodegenerative diseases: lessons from frontotemporal dementia and amyotrophic lateral sclerosis. Neurobiol. Dis. 154, 105360. 10.1016/j.nbd.2021.105360 33812000 PMC8113138

[B85] Sáez J. J. Diaz J. Ibañez J. Bozo J. P. Cabrera Reyes F. Alamo M. (2019). The exocyst controls lysosome secretion and antigen extraction at the immune synapse of B cells. J. Cell Biol. 218, 2247–2264. 10.1083/jcb.201811131 31197029 PMC6605794

[B86] Saffi G. T. Botelho R. J. (2019). Lysosome fission: planning for an exit. Trends Cell Biol. 29, 635–646. 10.1016/j.tcb.2019.05.003 31171420

[B87] Sanfridson A. Hester S. Doyle C. (1997). Nef proteins encoded by human and simian immunodeficiency viruses induce the accumulation of endosomes and lysosomes in human T cells. Proc. Natl. Acad. Sci. 94, 873–878. 10.1073/pnas.94.3.873 9023349 PMC19606

[B88] Sawa-Makarska J. Baumann V. Coudevylle N. von Bülow S. Nogellova V. Abert C. (2020). Reconstitution of autophagosome nucleation defines Atg9 vesicles as seeds for membrane formation. Science 369, eaaz7714. 10.1126/science.aaz7714 32883836 PMC7610778

[B89] Scerra G. De Pasquale V. Scarcella M. Caporaso M. G. Pavone L. M. D'Agostino M. (2022). Lysosomal positioning diseases: beyond substrate storage. Open Biol. 12, 220155. 10.1098/rsob.220155 36285443 PMC9597170

[B90] Scheuring D. Viotti C. Krüger F. Künzl F. Sturm S. Bubeck J. (2011). Multivesicular bodies mature from the trans-Golgi network/early endosome in Arabidopsis. Plant Cell 23, 3463–3481. 10.1105/tpc.111.086918 21934143 PMC3203422

[B91] Scotto Rosato A. Krogsaeter E. K. Jaslan D. Abrahamian C. Montefusco S. Soldati C. (2022). TPC2 rescues lysosomal storage in mucolipidosis type IV, Niemann-Pick type C1, and Batten disease. EMBO Mol. Med. 14, e15377. 10.15252/emmm.202115377 35929194 PMC9449600

[B92] Seto S. Tsujimura K. Koide Y. (2011). Rab GTPases regulating phagosome maturation are differentially recruited to mycobacterial phagosomes. Traffic 12, 407–420. 10.1111/j.1600-0854.2011.01165.x 21255211

[B93] Shammas H. Kuech E.-M. Rizk S. Das A. M. Naim H. Y. (2019). Different niemann-pick C1 genotypes generate protein phenotypes that vary in their intracellular processing, trafficking and localization. Sci. Rep-Uk 9, 5292. 10.1038/s41598-019-41707-y PMC6438969 30923329

[B94] Shaughnessy L. M. Hoppe A. D. Christensen K. A. Swanson J. A. (2006). Membrane perforations inhibit lysosome fusion by altering pH and calcium in Listeria monocytogenes vacuoles. Cell Microbiol. 8, 781–792. 10.1111/j.1462-5822.2005.00665.x 16611227 PMC1435990

[B95] Shelke G. V. Williamson C. D. Jarnik M. Bonifacino J. S. (2023). Inhibition of endolysosome fusion increases exosome secretion. J. Cell Biol. 222, e202209084. 10.1083/jcb.202209084 37213076 PMC10202829

[B96] Shin Y. H. Lee S. J. Jung J. (2012). Secretion of ATP from Schwann cells through lysosomal exocytosis during Wallerian degeneration. Biochem. Biophysical Res. Commun. 429, 163–167. 10.1016/j.bbrc.2012.10.121 23142593

[B97] Sho T. Li Y. Jiao H. Yu L. (2024). Migratory autolysosome disposal mitigates lysosome damage. J. Cell Biol. 223, e202403195. 10.1083/jcb.202403195 39347717 PMC11457477

[B98] Shtuhin-Rahav R. Olender A. Zlotkin-Rivkin E. Bouman E. A. Danieli T. Nir-Keren Y. (2023). Enteropathogenic *E. coli* infection co-elicits lysosomal exocytosis and lytic host cell death. mBio 14, e0197923. 10.1128/mbio.01979-23 38038448 PMC10746156

[B99] Sivaramakrishnan V. Bidula S. Campwala H. Katikaneni D. Fountain S. J. (2012). Constitutive lysosome exocytosis releases ATP and engages P2Y receptors in human monocytes. J. Cell Sci. 125, 4567–4575. 10.1242/jcs.107318 22767503

[B100] Skowyra M. L. Schlesinger P. H. Naismith T. V. Hanson P. I. (2018). Triggered recruitment of ESCRT machinery promotes endolysosomal repair. Science 360, eaar5078. 10.1126/science.aar5078 29622626 PMC6195421

[B101] Solvik T. A. Nguyen T. A. Tony Lin Y. H. Marsh T. Huang E. J. Wiita A. P. (2022). Secretory autophagy maintains proteostasis upon lysosome inhibition. J. Cell Biol. 221, e202110151. 10.1083/jcb.202110151 35446347 PMC9036093

[B102] Sou Y. S. Yamaguchi J. Masuda K. Uchiyama Y. Maeda Y. Koike M. (2024). Golgi pH homeostasis stabilizes the lysosomal membrane through N-glycosylation of membrane proteins. Life Sci. Alliance 7, e202402677. 10.26508/lsa.202402677 39079741 PMC11289521

[B103] Stinchcombe J. Bossi G. Griffiths G. M. (2004). Linking albinism and immunity: the secrets of secretory lysosomes. Science 305, 55–59. 10.1126/science.1095291 15232098

[B104] Sun S. Zhao G. Jia M. Jiang Q. Li S. Wang H. (2024). Stay in touch with the endoplasmic reticulum. Sci. China Life Sci. 67, 230–257. 10.1007/s11427-023-2443-9 38212460

[B105] Tan J. X. Finkel T. (2022). A phosphoinositide signalling pathway mediates rapid lysosomal repair. Nature 609, 815–821. 10.1038/s41586-022-05164-4 36071159 PMC9450835

[B106] Tan J. X. Finkel T. (2023). Lysosomes in senescence and aging. EMBO Rep. 24, e57265. 10.15252/embr.202357265 37811693 PMC10626421

[B107] Thakur R. S. O’Connor-Giles K. M. (2023). PDZD8 promotes autophagy at ER-Lysosome contact sites to regulate synaptogenesis. bioRxiv 2023. 10.1101/2023.10.30.564828 PMC12180128 40156832

[B108] Thayer D. A. Jan Y. N. Jan L. Y. (2013). Increased neuronal activity fragments the Golgi complex. Proc. Natl. Acad. Sci. U. S. A. 110, 1482–1487. 10.1073/pnas.1220978110 23297202 PMC3557034

[B109] Tsunemi T. Perez-Rosello T. Ishiguro Y. Yoroisaka A. Jeon S. Hamada K. (2019). Increased lysosomal exocytosis induced by lysosomal Ca(2+) channel agonists protects human dopaminergic neurons from α-synuclein toxicity. J. Neurosci. 39, 5760–5772. 10.1523/JNEUROSCI.3085-18.2019 31097622 PMC6636071

[B110] Udayar V. Chen Y. Sidransky E. Jagasia R. (2022). Lysosomal dysfunction in neurodegeneration: emerging concepts and methods. Trends Neurosci. 45, 184–199. 10.1016/j.tins.2021.12.004 35034773 PMC8854344

[B111] van Vliet A. R. Chiduza G. N. Maslen S. L. Pye V. E. Joshi D. De Tito S. (2022). ATG9A and ATG2A form a heteromeric complex essential for autophagosome formation. Mol. Cell 82, 4324–4339.e8. 10.1016/j.molcel.2022.10.017 36347259

[B112] Vitry S. Bruyere J. Hocquemiller M. Bigou S. Ausseil J. Colle M. A. (2010). Storage vesicles in neurons are related to Golgi complex alterations in mucopolysaccharidosis IIIB. Am. J. Pathol. 177, 2984–2999. 10.2353/ajpath.2010.100447 21037080 PMC2993280

[B113] Walia K. Sharma A. Paul S. Chouhan P. Kumar G. Ringe R. (2024). SARS-CoV-2 virulence factor ORF3a blocks lysosome function by modulating TBC1D5-dependent Rab7 GTPase cycle. Nat. Commun. 15, 2053. 10.1038/s41467-024-46417-2 38448435 PMC10918171

[B114] Wang F. Gómez-Sintes R. Boya P. (2018). Lysosomal membrane permeabilization and cell death. Traffic 19, 918–931. 10.1111/tra.12613 30125440

[B115] Wang F. Peters R. Jia J. Mudd M. Salemi M. Allers L. (2023). ATG5 provides host protection acting as a switch in the atg8ylation cascade between autophagy and secretion. Dev. Cell 58, 866–884.e8. 10.1016/j.devcel.2023.03.014 37054706 PMC10205698

[B116] Wang T. Hong W. (2002). Interorganellar regulation of lysosome positioning by the Golgi apparatus through Rab34 interaction with Rab-interacting lysosomal protein. Mol. Biol. Cell 13, 4317–4332. 10.1091/mbc.e02-05-0280 12475955 PMC138636

[B117] Wang X. Xu P. Bentley-DeSousa A. Hancock-Cerutti W. Cai S. Johnson B. T. (2024). Lysosome damage triggers acute formation of ER to lysosomes membrane tethers mediated by the bridge-like lipid transport protein VPS13C. bioRxiv. 10.1101/2024.06.08.598070

[B118] Wünkhaus D. Tang R. Nyame K. Laqtom N. N. Schweizer M. Scotto Rosato A. (2024). TRPML1 activation ameliorates lysosomal phenotypes in CLN3 deficient retinal pigment epithelial cells. Sci. Rep-Uk 14, 17469. 10.1038/s41598-024-67479-8 PMC11289453 39080379

[B119] Wyant G. A. Abu-Remaileh M. Frenkel E. M. Laqtom N. N. Dharamdasani V. Lewis C. A. (2018). NUFIP1 is a ribosome receptor for starvation-induced ribophagy. Science 360, 751–758. 10.1126/science.aar2663 29700228 PMC6020066

[B120] Xie Y. X. Naseri N. N. Fels J. Kharel P. Na Y. Lane D. (2022). Lysosomal exocytosis releases pathogenic α-synuclein species from neurons in synucleinopathy models. Nat. Commun. 13, 4918. 10.1038/s41467-022-32625-1 35995799 PMC9395532

[B121] Xu Y. Du S. Marsh J. A. Horie K. Sato C. Ballabio A. (2021). TFEB regulates lysosomal exocytosis of tau and its loss of function exacerbates tau pathology and spreading. Mol. Psychiatry 26, 5925–5939. 10.1038/s41380-020-0738-0 32366951 PMC7609570

[B122] Yim W. W. Yamamoto H. Mizushima N. (2022). Annexins A1 and A2 are recruited to larger lysosomal injuries independently of ESCRTs to promote repair. FEBS Lett. 596, 991–1003. 10.1002/1873-3468.14329 35274304

[B123] Zhang J. Kennedy A. de Melo Jorge D. M. Xing L. Reid W. Bui S. (2024). SARS-CoV-2 remodels the Golgi apparatus to facilitate viral assembly and secretion. bioRxiv, 10.1101/2022.03.04.483074 PMC12208438 40540531

[B124] Zhang K. R. Jankowski C. S. R. Marshall R. Nair R. Más Gómez N. Alnemri A. (2023). Oxidative stress induces lysosomal membrane permeabilization and ceramide accumulation in retinal pigment epithelial cells. Dis. Models and Mech. 16, dmm050066. 10.1242/dmm.050066 PMC10399446 37401371

[B125] Zhang Z. Chen G. Zhou W. Song A. Xu T. Luo Q. (2007). Regulated ATP release from astrocytes through lysosome exocytosis. Nat. Cell Biol. 9, 945–953. 10.1038/ncb1620 17618272

[B126] Zhitomirsky B. Assaraf Y. G. (2017). Lysosomal accumulation of anticancer drugs triggers lysosomal exocytosis. Oncotarget 8, 45117–45132. 10.18632/oncotarget.15155 28187461 PMC5542171

[B127] Zhong D. Wang R. Zhang H. Wang M. Zhang X. Chen H. (2023). Induction of lysosomal exocytosis and biogenesis via TRPML1 activation for the treatment of uranium-induced nephrotoxicity. Nat. Commun. 14, 3997. 10.1038/s41467-023-39716-7 37414766 PMC10326073

